# A Comparison of the Interstitial and Blood Glucose Responses Following Consumption of Different Carbohydrate-Containing Beverages in Humans: A Randomised Controlled Trial

**DOI:** 10.3390/nu18122033

**Published:** 2026-06-22

**Authors:** Ross Hamilton, Stephen C. Bain, Richard M. Bracken

**Affiliations:** 1 Applied Sport, Technology, Exercise and Medicine Research Centre, Faculty of Science and Engineering, Swansea University, Swansea SA1 8EN, UK; 2 Medical School, Swansea University, Swansea SA1 8EN, UK; 3 Health Technology and Solutions Interdisciplinary Research Institute, Faculty of Science and Engineering, Swansea University, Swansea SA1 8EN, UK

**Keywords:** continuous glucose monitoring, carbohydrate, blood glucose

## Abstract

Objectives: This study investigated the relationship between interstitial and blood glucose concentrations following ingestion of carbohydrate-containing drinks differing in carbohydrate amount, osmolarity, and glycaemic index. Methods: Ten healthy adults (nine male; age: 22 ± 1 y; height: 177 ± 12 cm; weight: 75 ± 14 kg) completed a double-blind, randomised cross-over study with seven beverage trials varying in carbohydrate (CHO) characteristics. Blood samples were collected at rest and over two hours, while interstitial glucose ([iG]) was recorded using a continuous glucose monitor (Supersapiens, Atlanta, USA). Glycaemic metrics and mean absolute relative difference (MARD) were calculated for hypoglycaemic, euglycaemic, and hyperglycaemic ranges. Data were analysed using repeated-measures ANOVA with Bonferroni correction and paired *t*-tests (*p* ≤ 0.05). Results: Interstitial and blood glucose concentrations were similar at baseline but diverged post-ingestion. MARD varied by glucose rate and direction, exceeding 20% during rapid declines (>2 mg/dL/min), where [iG] underestimated [BG] by −7.3 ± 27.1 mg/dL. Accuracy was highest during stable glucose (MARD = 10.5 ± 8.6%). Carbohydrate amount and glycaemic index influenced peak glucose, whereas beverage concentration (5–20%) had minimal effect when CHO amount was fixed, though variation in CGM agreement appeared during post-peak declines. Conclusions: CGM tracked blood glucose overall but showed reduced accuracy during rapid falls or hypoglycaemia. Carbohydrate properties influenced glycaemic response but not sensor agreement when CHO load was constant. Glucose rate and direction of change are key considerations for interpreting CGM data in research and applied settings.

## 1. Introduction

Continuous interstitial glucose monitoring (CGM) has gained considerable interest within the athletic and wellness communities as a surrogate measure of blood glucose (BG). Originally developed for diabetes management, CGM now offers individuals without diabetes 24 h glucose profiles, capturing fluctuations around meals, exercise, and sleep Bowler, Whitfield [1], Klonoff, Nguyen [2]. These insights can help identify how meal composition, size, and timing influence glycaemia [3] and guide good glycaemic behaviours.

CGM measures glucose concentration in the interstitial fluid, a compartment whose volume is dynamic [4]. Further, hydration status, exercise, and carbohydrate intake influence the movement of glucose between blood and interstitial fluid, creating differences in concentration between compartments [5,6]. Ingested carbohydrates vary in their rate of appearance into the circulation due to the amount and type; responses are further shaped by inter-individual characteristics [7,8]. These physiological influences can alter the interstitial glucose-to-blood (iG:BG) relationship, meaning that, even with modern algorithms, interstitial readings would be expected to deviate from real-time blood glucose values.

Small but consistent differences exist between iG and BG and are well documented, even within manufacturer guidelines for use. This is especially noted during exercise [9,10]. CGM accuracy, commonly expressed as mean absolute relative difference (MARD), varies across conditions, with modern devices showing values from ~10–20% depending on physiological state [11]. Exercise represents one condition in which MARD can be substantially elevated [12], reflecting exercise-induced physiological changes including plasma volume shifts, alterations in hydration status, and changes in hepatic glucose release and muscular glucose uptake. These changes can complicate the real-time interpretation of CGM-derived glucose trends.

Understanding additional factors (particularly at rest) may provide important information that might add to the confidence and accuracy during other dynamic physiological processes such as feeding, particularly in acute settings, where it may inform nutrient intake decision-making in people without diabetes. However, the extant research in this area is limited.

Recent research examining the carbohydrate content of meals showed that the magnitude of the postprandial glycaemic response corresponds to the proportion of carbohydrate in the meal [13]. Baird et al. [14] demonstrated that glycaemic load could be manipulated through reductions in glycaemic carbohydrate as well as increases in protein and fat in a milk beverage [14], with these manipulations verifiable through the use of CGM. However, some concerns have been raised on the ability of CGM to accurately reflect the glycaemic index of different meals, with some overestimation being reported [8]. The accuracy of CGM in response to the ingestion of glucose in differing solutions has also been highlighted as a concern, where both physiological delay and random discrepancies may be present [15]. Research has yet to adequately examine how osmolarity and carbohydrate amount alone or in combination shape our understanding of the interstitial–blood glucose relationship.

The present study, therefore, aimed to investigate the relationship between interstitial and blood glucose concentrations following ingestion of different carbohydrate-containing drinks differing in carbohydrate amount, osmolarity, and glycaemic index.

## 2. Methods

### 2.1. Participants and Ethical Approval

A total of 11 healthy adults (10 male, age: 22 ± 1 years, height: 177 ± 12 cm, weight: 75 ± 14 kg) took part in this double-blind, randomised, counterbalanced study. Ethical approval was granted by the Swansea University Research Ethics Committee. The study was carried out in accordance with the Declaration of Helsinki and the International Conference on Harmonisation of Good Clinical Practice. All volunteers provided written informed consent prior to study involvement. Before undertaking any experimental procedures, participants completed a screening visit during which eligibility for trial inclusion was assessed alongside a review of their medical history via the PAR-Q questionnaire. After confirmation of study suitability, data on anthropometric characteristics were collected and a familiarization session with the blood sampling procedure was completed.

### 2.2. Study Design

**Figure 1:**
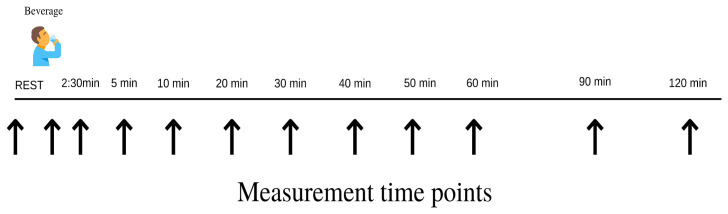
Study timeline showing consumption of beverage and measurement timepoints thereafter.

This study used a randomised, double-blind, cross-over design to investigate the impact of the glycaemic index, amount of CHO, and concentration of a CHO-containing beverage. The trial day schedule of measurements is displayed in Figure 1 below.

Following randomised allocation to each sub-aim trial (via computerised randomising sequence software) participants completed seven experimental trials. Trial beverages were prepared by a laboratory assistant outside of the research team to ensure double-blinding. Drinks were formulated and labelled with specific codes, also assigned using an online randomiser software (www.randomizer.org). Identification of each trial beverage was only made available to the research team on cessation of study trials.

On each day of the trial, participants would attend a laboratory trial session having applied a CGM sensor a minimum of 24 h prior. They then consumed their assigned beverage and remained in a seated position for the following 2 h, while measurements were taken at fixed timepoints (see Figure 1).

### 2.3. Beverage Formulation

Beverages were formulated using isomaltulose (PalatinoseTM) (ISO) (BENEO, Mannheim, Germany) or dextrose (Bulk powders, Colchester, UK). Each beverage had approximately 10 mL of flavoured cordial (Britvic PLC, Hemel Hempstead, UK), which added approximately 2 mg of additional carbohydrate to each beverage. Three separate beverage comparisons were tested. Carbohydrate amount: 50 g dextrose (1030 mOsm·kgH_2_O), 25 g dextrose (720 mOsm·kgH_2_O), and 10 g dextrose (360 mOsm·kgH_2_O), each in 500 mL water. Carbohydrate concentration: 5% (25 g/500 mL; 720 mOsm·kgH_2_O), 10% (25 g/250 mL; 1270 mOsm·kgH_2_O), and 20% (25 g/125 mL; 2540 mOsm·kgH_2_O). Glycaemic index: high-GI dextrose (50 g/500 mL; 1030 mOsm·kgH_2_O), low-GI isomaltulose (50 g/500 mL; 970 mOsm·kgH_2_O), and placebo (500 mL; 73 mOsm·kgH_2_O).

### 2.4. Collection of Interstitial Glucose Concentration

All interstitial glucose [iG] data were recorded via the Abbott Libre Sense Biosensor (Abbott Laboratories, Chicago, IL, USA). The CGM device was paired to the Supersapiens receiver and Software application (TT1 Products Inc., Atlanta, GA, USA), which was installed on the participant’s smartphone. Displayed point iG concentration values, representing discrete minute-by-minute readings rather than algorithmically smoothed trend data, were recorded simultaneously with blood collection from the smartphone display at each time point for later analysis via Microsoft Excel 2019 (Microsoft Corp., Redmond, WA, USA).

### 2.5. Assessment of CGM Accuracy

Mean absolute relative difference (MARD) was used to assess the accuracy of the CGM sensor’s [iG] against BG. The absolute relative difference (in percent) reflects the difference between the two measurements. This was calculated for overall MARD as well as during different target ranges: hypoglycaemia (<70 mg/dL, a biochemical threshold rather than a clinical diagnosis in this healthy cohort), euglycaemia (71–140 mg/dL), and hyperglycaemia (>140 mg/dL). The MARD was also assessed during different rates of concentration change: ‘Quick’ rate of change (>2 mg/dL/min), ‘Changing’ (1–2 mg/dL/min), and ‘Slow’ rate of change (<1 mg/dL/min). Surveillance error grids are provided for all measured pairs as well as pairs within each glucose threshold range as per Klonoff et al. [2].

A Bland–Altman plot was used to assess the systematic measurement differences between blood and interstitial glucose values. This analysis provides directional insights into any biases that may appear between the measurement compartments. The 95% limits of agreement were calculated as the mean difference ± 1.96 times the standard deviation [16].

### 2.6. Blood Sampling

Capillary blood samples were taken from the fingertip. The sampling site was prepared by cleaning with an alcohol swab before using a lancet to collect the blood sample which was analysed for glucose and lactate (Biosen, EFK, Bremen, Germany). An additional sample was also analysed for haemoglobin (Haemocue, Ängelholm, Sweden) and haematocrit (Hawksley reader, Sussex, UK) following centrifugation. These were used for the estimation of plasma volume changes as per Dill and Costill [17].

### 2.7. Statistical Analyses

Statistical analyses were carried out using Excel (Microsoft Office) and Graphpad Prism V 9.5 (GraphPad Software, Inc. San Diego, CA, USA). All data are presented as the mean ± standard deviation (SD). Data were analysed using one- or two-way ANOVAs with Bonferroni adjustment and paired *t*-tests where appropriate. Significant differences were reported if *p* ≤ 0.05. Sphericity was not assumed and the Greenhouse-Geisser correction was applied to all repeated-measures ANOVAs. Where CGM data continuity was interrupted, affected trials were excluded to maintain data integrity; as a result, analysed *n* varies by condition (range 9–11).

## 3. Results

Where CGM data continuity was interrupted, affected trials were excluded to maintain data integrity; analysed n therefore varies by condition (range 9–11).

### 3.1. Glycaemic Responses

#### 3.1.1. Carbohydrate Amount

**Figure 2:**
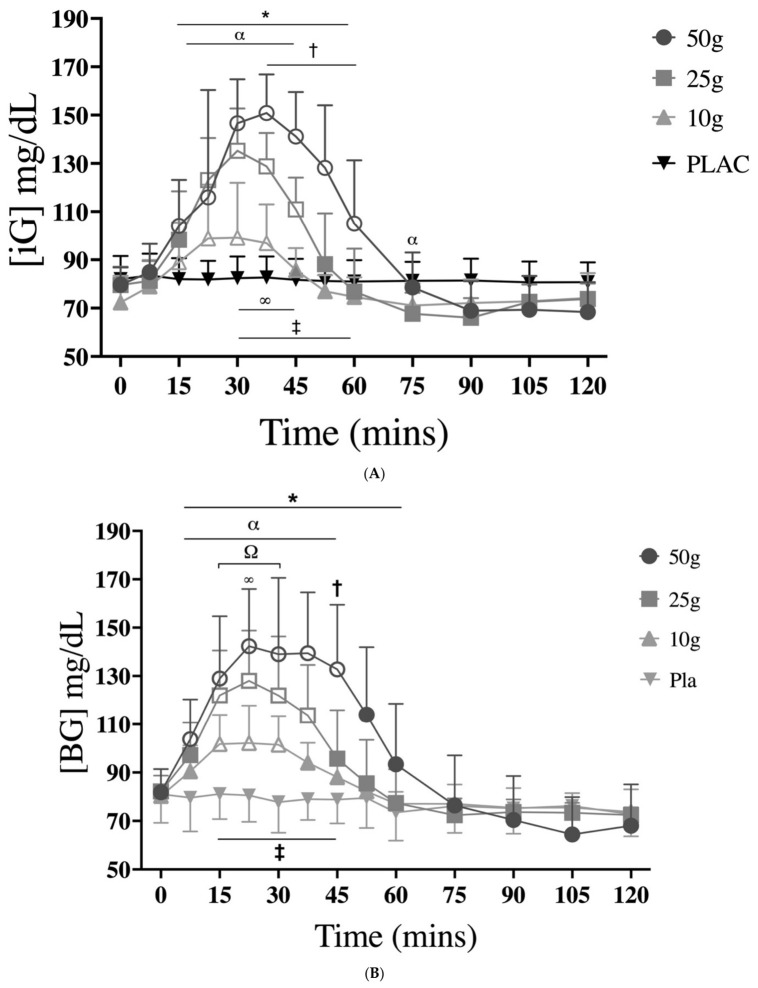

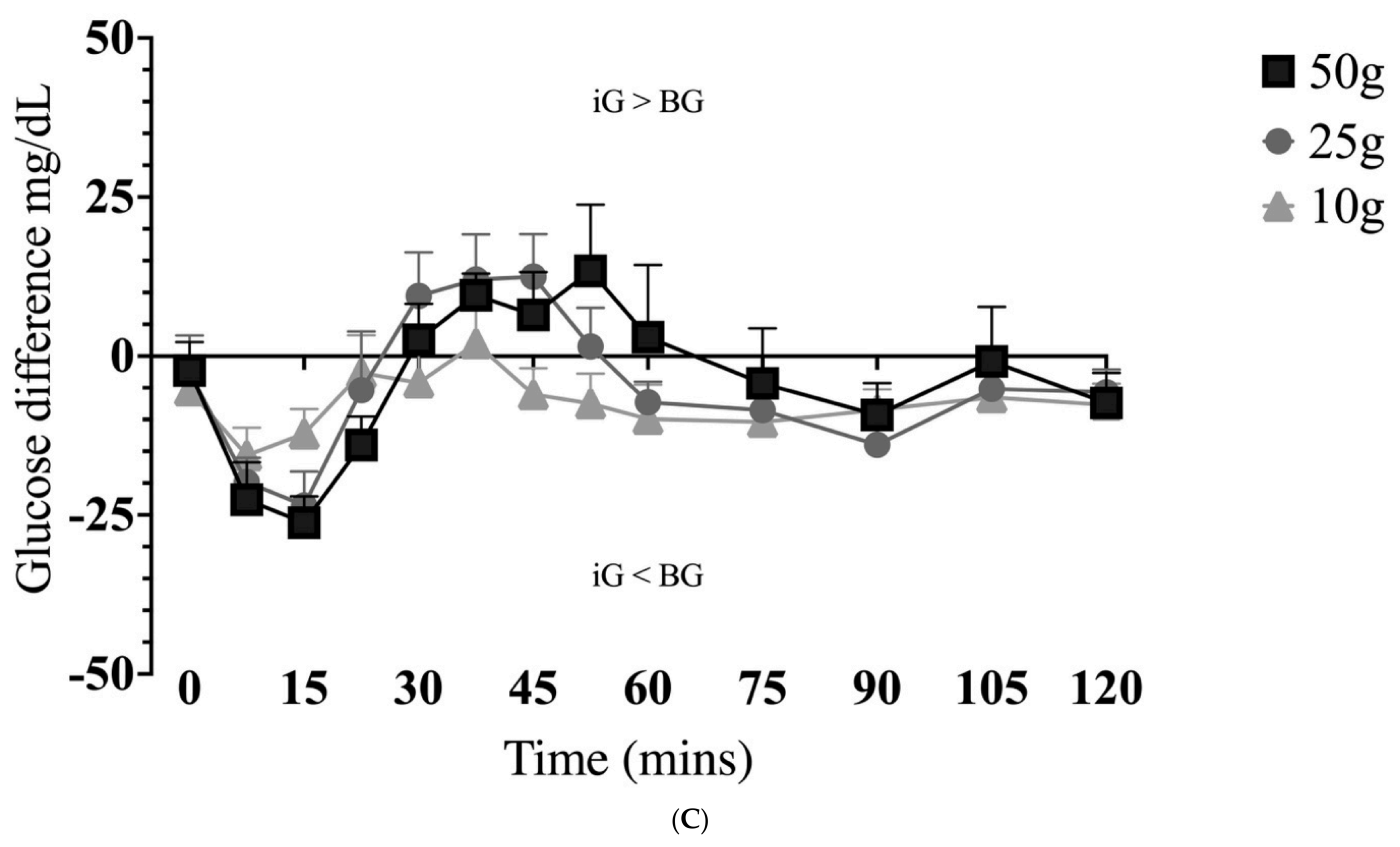
(**A**) Interstitial glucose concentration [iG], (**B**) blood glucose concentrations [BG], and (**C**) the difference between [iG] and [BG] values in the carbohydrate-containing trials adjusted from the difference between [iG] and [BG] in PLAC (C is displayed as Mean ± SEM) following an overnight fast and over 2 h following ingestion of 50 g (*n* = 10), 25 g (*n* = 9), 10 g (*n* = 10), or Placebo (PLAC) (*n* = 11) of dextrose. Negative values indicate [iG] was lower than [BG], while positive values indicate where it was greater. All carbohydrates were consumed in 480 mL water with 20 mL flavouring (PLAC). Hollow sample points indicate changes from rest within the condition. * indicates a difference between 50 g and PLAC trial, † indicates a difference between 50 g and 25 g, and ‡ indicates differences between 50 g and 10 g. α indicates a difference between 25 g and PLAC. ∞ indicates a difference between 25 g and 10 g. Ω indicates a difference between 10 g and PLAC (*p* ≤ 0.05). Data are displayed as the mean ± SD; *n* varies by condition (range 9–11).

The glycaemic responses to differing amounts (50 g, 25 g, 10 g, and 0 g [PLAC]) of carbohydrate (dextrose) in 500 mL of water are displayed for [BG] and [iG] in Figure 2A and Figure 2B, respectively. A comparison of [iG] and [BG] measurements are displayed in Table 1.

#### 3.1.2. Influence of Carbohydrate Administration in Different Amounts on Interstitial Glucose Concentrations

At rest [iG] was similar between all beverages. From rest, the rate of change to peak was different in all beverages compared to PLAC (50 g +2.2 ± 0.6, 25 g +1.9 ± 0.5, 10 g +1.2 ± 0.7 vs. PLAC +0.1 ± 0.3 mg/dL/min, *p* < 0.05) and quicker when 50 g was compared to 10 g (*p* = 0.004). Peak [iG] was greater in all beverages compared to PLAC and larger amounts of carbohydrate displayed larger peaks (50 g 158 ± 17, 25 g 137 ± 17 10 g 107 ± 22 vs. PLAC 86 ± 8 mg/dL, *p* < 0.05). Moreover, 50 g was also greater than the peak in 10 g (*p* < 0.001). The time of peaks was similar in all beverages. The rate of change from the peak to minimum [iG] was different in all beverages compared to PLAC (50 g −1.8 ± 0.8, 25 g −1.6 ± 0.4 and 10 g −1.2 ± 0.8 vs. PLAC 0.0 ± 0.4 mg/dL/min, *p* < 0.05). The minimum [iG] was lower than PLAC in all beverages (50 g 62 ± 7, 25 g 62 ± 6, 10 g 68 ± 8 vs. PLAC 77 ± 8 mg/dL, *p* < 0.05). Mean [iG] was only greater than PLAC for the 50 g beverage (50 g 104 ± 9 vs. PLAC 82 ± 8 mg/dL, *p* < 0.001).

#### 3.1.3. Influence of Carbohydrate Administration in Different Amounts on Blood Glucose Concentrations

At rest [BG] was similar between all beverages. From rest, the rate of change to peak was different for all beverages when they were compared to PLAC (50 g +2.3 ± 0.1, 25 g +2.3 ± 0.8 and 10 g +1.3 ± 0.7 vs. PLAC +0.2 ± 0.4 mg/dL/min, *p* < 0.05). Peak [BG] was also greater in all beverages when compared to PLAC (50 g 154 ± 23, 25 g 133 ± 17 and 10 g 107 ± 14 vs. PLAC 84 ± 12 mg/dL, *p* < 0.05). Peaks were greater in both 50 g and 25 g when compared to 10 g (*p* < 0.05). The timing of the peak [BG] was similar with all beverages. The rate of change from peak to minimum [BG] was different with both 50 g and 25 g compared to PLAC (50 g −1.3 ± 0.4 and 25 g −1.3 ± 0.4 vs. PLAC −0.4 ± 0.3 mg/dL/min, *p* < 0.05). Minimum [BG] and the timing of the minimum similar between all beverages, occurring between approximately 85 and 102 min. Finally, mean [BG] was greater with all beverages than PLAC (50 g 104 ± 13, 25 g 94 ± 10, and 10 g 85 ± 8 vs. PLAC 77 ± 10 mg/dL, *p* < 0.05). The mean [BG] was greater in 50 g compared to the 10 g (*p* = 0.002).

#### 3.1.4. Comparison of Blood and Interstitial Glucose Concentrations in Response to Consumption of Different Amounts of Carbohydrate

The difference between [iG] and [BG] across the 2 h period have been adjusted to account for the difference between [iG] and [BG] in the PLAC trial and displayed in Figure 2C. All differences are displayed as (Δ = [iG] − [BG]).

During the PLAC trial [iG] and [BG] glycaemic metrics were similar, apart from the higher [iG] minimum values (Δ+8.5 ± 11.5 mg/dL, *p* = 0.035). At rest in the 10 g trial, [iG] was lower than [BG] (Δ−8 ± 8.9 mg/dL, *p* = 0.027). The rate of change for [iG] and [BG] was similar in all beverages, although peak [iG] occurred later than [BG] during both the 25 g and 10 g trial [BG] (25 g Δ+8 ± 5, and 10 g Δ+5 ± 6 min, *p* < 0.05). Peaks were similar in both [iG] and [BG] for all beverages. In the 10 g trial, the rate of change from [iG] peak to minimum was slower (Δ−0.6 ± 0.7 mg/dL/min, *p* = 0.019) and the time of the [iG] minimum was earlier than [BG] (Δ30 ± 27 min, *p* = 0.010). The minimum [iG] was lower than [BG] in 25 g (Δ5.3 ± 5.7 mg/dL, *p* = 0.033). Mean [iG] and [BG] were similar in all beverages.

#### 3.1.5. The Concentration of Carbohydrate Solution

**Figure 3:**
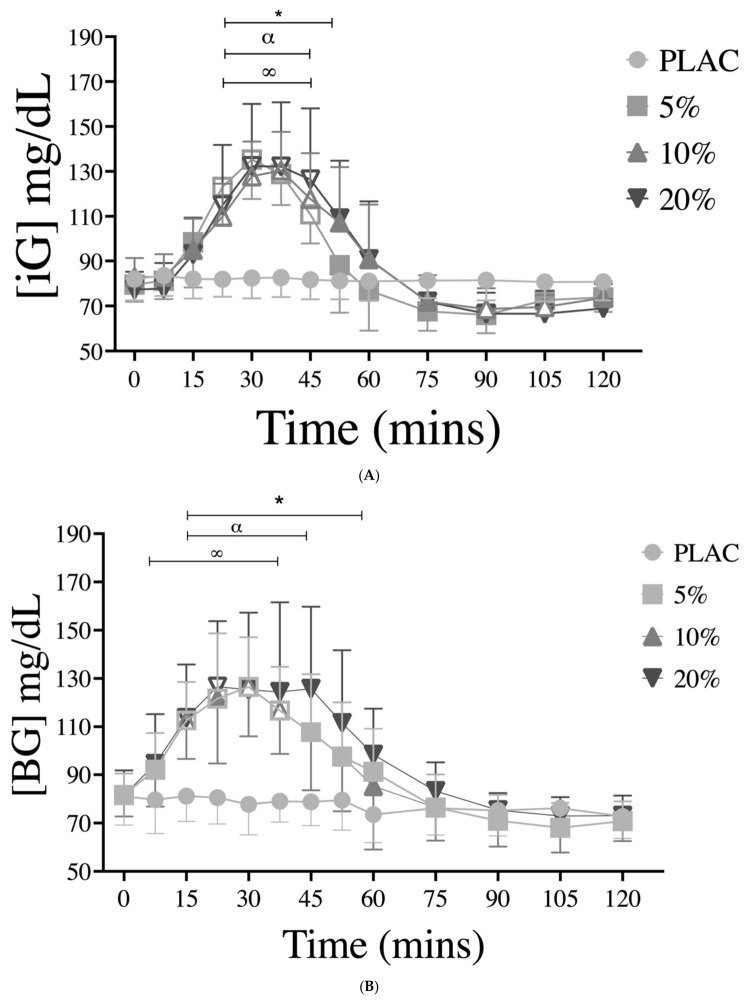

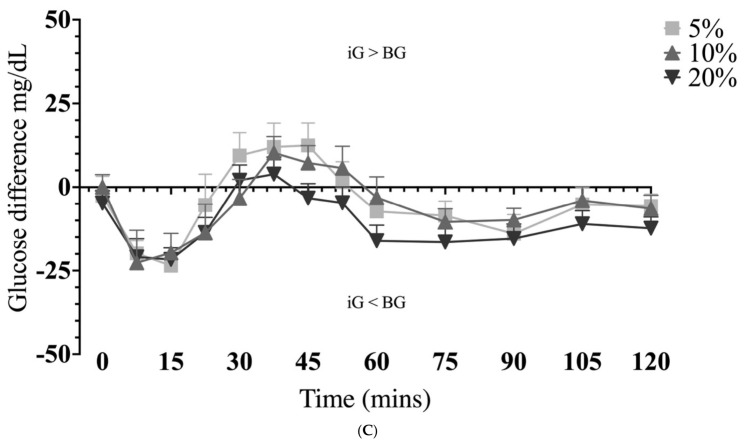
(**A**) Interstitial glucose concentration [iG], (**B**) blood glucose concentrations, and (**C**) the difference between [iG] and [BG] values in carbohydrate-containing beverages adjusted from the differences between [iG] and [BG] in the PLAC values (C is displayed as Mean ± SEM) following a 2 h post ingestion of a 20% solution of dextrose (20%) (*n* = 10), a 10% solution (10%) (*n* = 10), a 5% solution (*n* = 9), or Placebo (PLAC) (*n* = 11). Negative values indicate [iG] was lower than [BG], while positive values indicate where it was greater. All carbohydrates were consumed in water with 20 mL flavouring (PLAC). Hollow sample points indicate changes from rest within condition (*p* ≤ 0.05). * indicates differences between 20% and PLAC. α indicates a difference between 10% and PLAC. ∞ indicates a difference between 5% and PLAC (*p* ≤ 0.05). Data are displayed as the mean ± SD; *n* varies by condition (range 9–11).

The glycaemic responses to differing concentrations (20%, 10%, 5%, and a placebo [PLAC]) of carbohydrate (25 g of dextrose) in different amounts (500, 250, and 125 mls) of water are displayed for [iG] and [BG] in Figure 3A and Figure 3B, respectively. Detailed glycaemic metrics with a comparison of [iG] and [BG] measurements are displayed in Table 1.

#### 3.1.6. Influence of Different Carbohydrate Concentration Solutions on Interstitial Glucose Concentrations

At rest [iG] was similar with all beverages compared to the PLAC. From rest, the rate of change to peak was different with all beverages compared to PLAC (5% +1.9 ± 0.5, 10% +1.4 ± 0.6 and 20% +1.7 ± 0.7 mg/dL/min, *p* < 0.05). Peak [iG] was greater in all beverages when compared to PLAC (5% 137 ± 17, 10% 134 ± 18, and 20% 138 ± 26 mg/dL, *p* < 0.05). The time of the peak concentration was similar with all beverages, occurring between ~30–45 min. The rate of change from peak to minimum [iG] was different for all beverages compared to PLAC (5% −1.6 ± 0.4, 10% −1.4 ± 0.4 and 20% −1.6 ± 0.7 mg/dL/min, *p* < 0.05). The minimum [iG] was lower in all beverages when compared to PLAC (5% 63 ± 6, 10% 64 ± 5 and 20% 60 ± 3 mg/dL, *p* < 0.05) but the timing was similar occurring between approximately 70 and 90 min). Finally, mean [iG] was greater for both 10% and 20% than it was for PLAC (10% 94 ± 11 and 20% 94 ± 13 mg/dL, *p* < 0.05).

#### 3.1.7. Influence of Different Carbohydrate Concentration Solutions on Blood Glucose Concentrations

At rest [BG] was similar between all beverages. From rest, the rate of change to peak was different for all beverages when compared to PLAC (5% +2.3 ± 0.1, 10% +1.8 ± 0.8 and 20% +1.7 ± 0.8 mg/dL/min, *p* < 0.05). The peak [BG] was greater in all of the beverages when compared to PLAC (5% 133 ± 17, 10% 133 ± 16, and 20% 137 ± 31 mg/dL, *p* < 0.05), the time of peak came earlier in 5% than 20% (5% 23 ± 5 vs. 20% 35 ± 9 min, *p* = 0.023). The rate of change from peak to minimum [BG] was different for both 5% and 10% solutions compared to PLAC (5% −1.3 ± 0.8, and 10% −1.3 ± 0.8 mg/dL/min, *p* < 0.05). The minimum [BG] and its timings were similar with all beverages, occurring between ~84 and 94 min. Mean [BG] was greater with all beverages compared to PLAC (5% 94 ± 10, 10% 94 ± 10, and 20% 101 ± 16 mg/dL, *p* < 0.05).

#### 3.1.8. Comparison of Interstitial and Blood Glucose Concentrations Following Consumption of Carbohydrate Solutions of Different Concentrations

The difference between [iG] and [BG] across the 2 h period in the CHO-containing trials have been adjusted from the difference between [iG] and [BG] in the PLAC trial and displayed in Figure 3C.

During the PLAC trial [iG] and [BG] glycaemic metrics were similar apart from the higher [iG] minimum values (Δ+8.5 ± 11.5 mg/dL, *p* = 0.035). At rest [iG] and [BG] were similar for all of the beverages. The rate of change to peak was similar for both [iG] and [BG]. In both the 5% and 10% trials, the time of the peak came later for [iG] (5% Δ+8 ± 5 and 10% Δ+5 ± 6 min, *p* = 0.05). The rate of change from peak to minimum [iG] in the 20% was slower than [BG] (Δ−0.6 ± 0.5 mg/dL/min, *p* = 0.007). In the 5% and 20% trials, the minimum [iG] was lower than [BG] (5% Δ−5 ± 5 and 20% Δ−8 ± 5 mg/dL, *p* < 0.05). Finally, the mean [iG] was lower than [BG] for the 20% trial (Δ−6.1 ± 7.4 mg/dL, *p* = 0.030).

#### 3.1.9. Glycaemic Index of Carbohydrates

**Figure 4:**
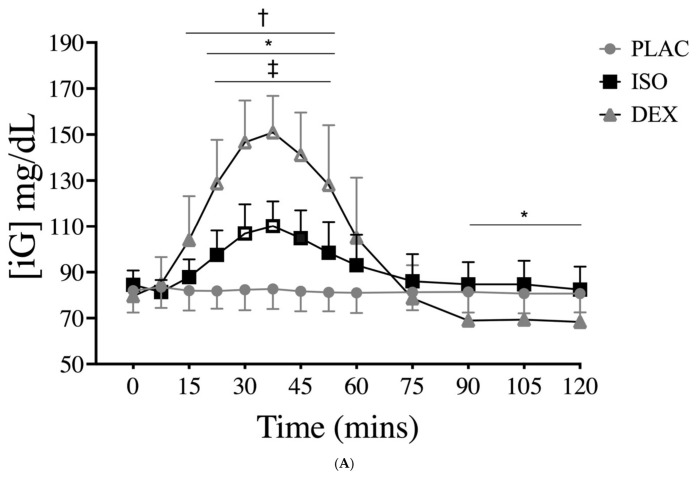

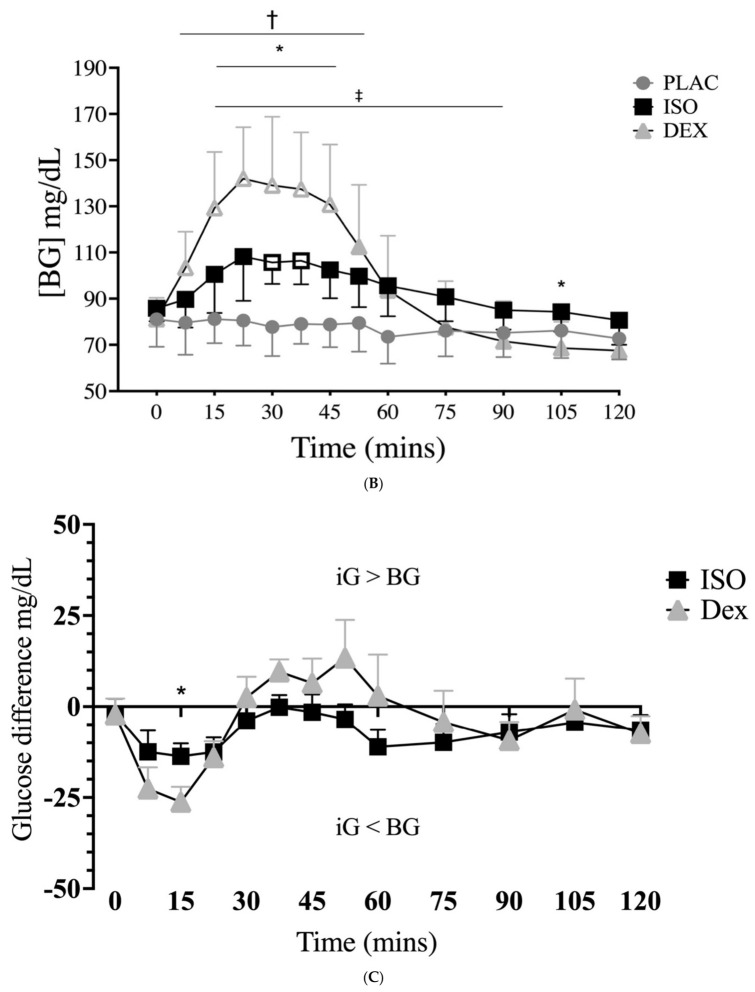
(**A**) Blood glucose concentrations [BG], (**B**) interstitial glucose concentrations [iG], and (**C**) the difference between [BG] and [iG] values adjusted from the PLAC values (C is displayed as Mean ± SEM) following an overnight fast and over a 2 h following ingestion of 50 g of Dextrose (DEX, *n* = 10), Isomaltulose (ISO, *n* = 10), or Placebo (PLAC, *n* = 11). All carbohydrates were consumed in 480 mL water with 20 mL flavouring (PLAC). Negative values indicate [iG] was lower than [BG], while positive values indicate where it was greater. Hollow sample points indicate changes from rest within condition. * indicates differences between DEX and ISO. † indicates differences between DEX and PLAC. ‡ indicates differences between ISO and PLAC (*p* ≤ 0.05). Data are displayed as the mean ± SD; *n* varies by condition (range 9–11).

The glycaemic responses to 50 g of carbohydrates with different glycaemic indices (Dextrose [DEX], Isomaltulose [ISO], and a Placebo [PLAC]) in 500 mL of water are displayed for [iG] and [BG] in Figure 4A and Figure 4B, respectively. Detailed glycaemic metrics with a comparison of [BG] and [iG] measurements are displayed in Table 1.

#### 3.1.10. Influence of Carbohydrate Glycaemic Index on Interstitial Glucose Concentrations

At rest [iG] was similar with all beverages. From rest, the rate of change to peak was different for both DEX and ISO compared to PLAC (ISO +0.7 ± 0.3 mg/dL/min, *p* < 0.005) and faster for DEX than ISO (*p* = 0.001). Peak [iG] was greater for both DEX and ISO compared to PLAC (ISO 113 ± 11 mg/dL, *p* < 0.05) but also greater in DEX than ISO (*p* < 0.001). The time of peak was similar with all trial beverages (~37–44 min). The rate of change from peak to minimum was different for both DEX and ISO than it was for the PLAC (ISO −0.7 ± 0.4 mg/dL/min, *p* < 0.05). The minimum [iG] was lower for DEX compared to PLAC (*p* < 0.001) and also lower than ISO (ISO 76 ± 7 mg/dL, *p* < 0.05), while the time of the minimum was similar for all trial beverages, occurring between ~70 and 98 min. Finally, the mean [iG] across the 2 h period was greater for both DEX and ISO compared to PLAC (ISO 93 ± 6 mg/dL, *p* = 0.05), and it was also greater in DEX compared to ISO (*p* = 0.01).

#### 3.1.11. Influence of Carbohydrate Glycaemic Index on Blood Glucose Concentrations

At rest [BG] was similar between all beverages. From rest, the rate of change to peak was different for DEX than PLAC (*p* < 0.001), and it was faster than ISO (+0.8 ± 0.1 mg/dL/min, *p* < 0.001). Peak [BG] was greater with both carbohydrate types compared to PLAC (ISO 113 ± 17 mg/dL, *p* < 0.05), and it was also greater in DEX than ISO (*p* < 0.001). The time of peak appeared later with ISO than it appeared with PLAC (ISO 42 ± 15 min, *p* = 0.039). The rate of change from peak to minimum was different with DEX than it was for PLAC (*p* = 0.001), and it was also faster than ISO (0.6 ± 0.4 mg/dL/min, *p* = 0.015). The minimum concentration and the timing of the minimums were similar for both DEX and ISO when compared to PLAC. DEX, however, displayed a lower minimum [BG] than ISO (78 ± 10 mg/dL, *p* = 0.012). Finally, the mean [BG] across the 2 h period was greater for both DEX and ISO compared to PLAC (ISO 95 ± 8 mg/dL, *p* < 0.05).

#### 3.1.12. Comparison of Blood and Interstitial Glucose Concentrations in Response to Consumption of Carbohydrate Differing in Glycaemic Index

The difference between [BG] and [iG] in the carbohydrate-containing beverages have been adjusted from the differences between the [iG] and PLAC trial and are displayed in Figure 4C.

During the PLAC trial, [iG] and [BG] metrics were similar with only minimum values being different (Δ8.5 ± 11.5 mg/dL, *p* = 0.035). At rest, [iG] and [BG] measurements were similar in both DEX and ISO. The rate of change to peak, the peaks, and the timing of the peaks were similar in both [iG] and [BG]. The rate of change from peak to minimum, the minimum, and its timing were all similar in both [iG] and [BG] in DEX and ISO. The mean [iG] and [BG] were also similar in both beverages.

##### CGM Accuracy

Across all paired samples (*n* = 923), the mean absolute relative difference (MARD) was 13.5 ± 13.8%, with a Bland–Altman bias of 1.1 ± 15.8 mg·dL^−1^ and 95% limits of agreement (LOA) from −29.9 to +32.0 mg·dL^−1^. During hyperglycaemia, bias increased to +15.8 ± 18.4 mg·dL^−1^ (LOA: −20.3 to +52.0 mg·dL^−1^), while in hypoglycaemia, MARD was 18.5 ± 25% and the CGM underestimated blood glucose by −6.9 ± 14.6 mg·dL^−1^ (LOA: −35.5 to +21.8 mg·dL^−1^). MARD was greater during hypoglycaemia than during euglycaemia (*p* < 0.001) and vs. overall (*p* < 0.001). Direction-specific patterns were also evident, with overestimation during rapid glucose rises (+12.9 ± 14.3 mg·dL^−1^; LOA: −15.3 to +40.8 mg·dL^−1^) and underestimation during rapid declines (−7.3 ± 27.1 mg·dL^−1^; LOA: −40.4 to +45.9 mg·dL^−1^). MARD values are displayed in Table 2 below.

### 3.2. Surveillance Error Grid

**Figure 5:**
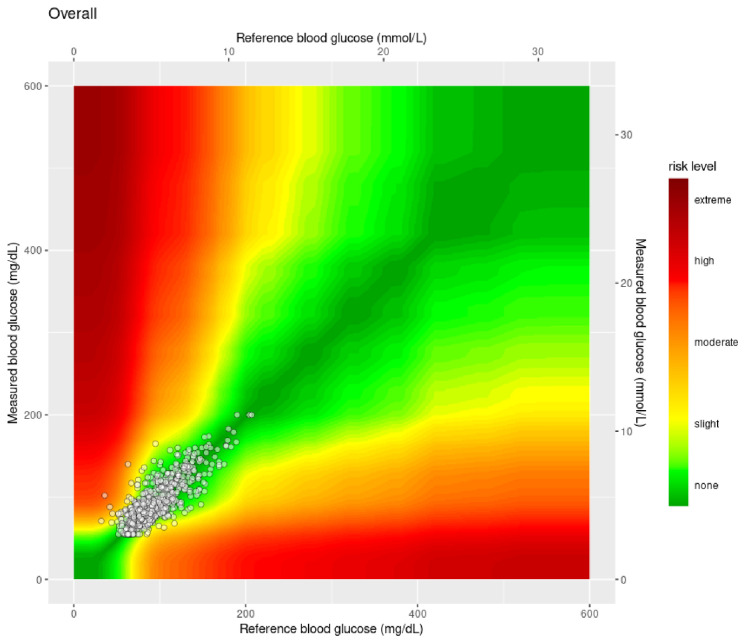
Colour-coded continuous surveillance error grid and risk levels for all glucose measurements comparing the continuous glucose monitoring (CGM) device to the reference measurement (901 pairs). Colours indicate associated risk levels ranging from none (dark green) to extreme (brown). Glycaemic range and during different rates of change as concentration rises and falls. All data are displayed as Mean ± SD (901 pairs).

Figure 5 below displays a surveillance error grid of all measured glucose data from the CGM paired to the reference measurements (901 pairs). A total of 72.4% (652 pairs) showed no risk of error, 22% (198) showed slight risk of lower measurement, 5.1% (46 pairs) showed slight risk of higher measurement, 0.4% (4 pairs) showed moderate risk of lower measurement, and 0.1% (1 pair) showed a moderate risk of overmeasurement.

### 3.3. Area Under the Curve (AUC)

Absolute AUC and placebo-adjusted (offset) AUC values are presented in Supplemental Table S1. Positive AUC refers to glucose excursions above baseline, while negative AUC reflects the area contained within the dips below baseline. The absolute AUC reflects overall glucose dynamics across the 2 h period while the offset values help to isolate the impacts of the individual beverage interventions. [iG] and [BG] in both absolute and offset values were similar only showing a difference in the 20% concentration values when offset against the placebo trial.

### 3.4. Plasma Volume Changes

Plasma volume changes from rest (baseline) were estimated using measures of haemoglobin and haematocrit at 30, 60, 90, and 120 min timepoints. Summary data for haemoglobin and haematocrit are displayed in Supplemental Table S2, while summary data for estimated plasma changes are displayed in Supplemental Table S3.

#### 3.4.1. Carbohydrate Amount

After consuming a beverage with different amounts of dextrose (50 g, 25 g, and 10 g) and a Placebo (PLAC) in a fixed volume of water (500 mL), no differences in haemoglobin changes were detected between amounts (*p* = 0.808). Differences were detected within the 25 g trial. Haemoglobin was different from baseline at 90 min (*p* = 0.05) and at 120 min (*p* = 0.023). Haematocrit was similar between carbohydrate amounts (*p* = 0.963) and within each trial across all timepoints (*p* = 0.999). Plasma volume changes were similar between amounts (*p* = 0.101) and within each trial across all timepoints (*p* = 0.925).

#### 3.4.2. Concentration of Solution

After consuming beverages containing a fixed amount of carbohydrate in differing fluid concentrations (5%, 10%, and 20%) no changes in haemoglobin were detected between solutions (*p* = 0.743). Differences were detected within the 10% trial. Haemoglobin was different from baseline at 90 min (*p* = 0.05) and at 120 min (*p* = 0.023). Haematocrit was similar between all solutions (*p* = 0.958). The timepoint of measurement affected haematocrit changes (*p* = 0.001); however, post hoc analysis did not locate differences within any specific trial. Plasma volume changes were similar between solutions (*p* = 0.262) and within each trial across all timepoints (*p* = 0.358).

#### 3.4.3. Glycaemic Index

After consuming 50 g of carbohydrates differing in their glycaemic index (DEX and ISO), haemoglobin plasma volume changes were similar between each carbohydrate type (*p* = 0.532) and within each trial across all timepoints (*p* = 0.229). Haematocrit was similar between each carbohydrate type (*p* = 0.825). Haematocrit changes were different between timepoints (*p* = 0.040), but post hoc analysis did not locate differences within either carbohydrate type. Plasma volume changes were similar between each carbohydrate type (*p* = 0.277) and within each trial across all timepoints (*p* = 0.852).

## 4. Discussion

This study aimed to investigate the relationship between interstitial and blood glucose concentrations following ingestion of various carbohydrate-containing beverages. By assessing the agreement between glycaemic metrics derived from both compartments, some potential physiological and sensor-related measurement differences were identified. The wide confidence limits observed indicate reduced accuracy of the sensor, particularly during periods of falling glucose and at lower concentrations. These findings highlight conditions that may affect CGM accuracy and the susceptibility of certain metrics to divergence from blood glucose measures.

### 4.1. Glycaemic Responses and Sensor Agreement

Overall CGM accuracy metrics, stratified by glycaemic state and rate of change, are summarised in Table 2 and form the basis of the accuracy discussion that follows. Overnight fasted, morning rested interstitial and blood glucose concentrations were similar at the start of all trials. Following ingestion of different carbohydrate-containing beverages, both [iG] and [BG] rose. The rate of glucose rise from baseline to peak was delayed for [iG] in several trials (Table 1). MARD values varied depending on the speed and direction of glucose change. While peak concentrations were generally similar, bias and error increased at higher glucose levels and during more dynamic phases of the OGTT. In agreement with other work, higher glucose concentrations and faster rates of change were associated with greater bias and reduced accuracy [18,19]. Across all paired samples (*n* = 923), MARD was 13.5 ± 13.8%, with a Bland–Altman bias of 1.1 ± 15.8 mg·dL^−1^ and 95% limits of agreement ranging from −29.9 to +32.0 mg·dL^−1^, indicating moderate overall agreement but considerable individual variability. The ± 30 mg·dL^−1^ limits indicate that despite low mean bias, individual readings could differ meaningfully between compartments, particularly during rapid glucose change. During periods of rising glucose, particularly >2 mg·dL^−1^·min^−1^, MARD increased to 13.8 ± 13.1%, with [iG] showing a positive bias (+12.9 ± 14.3 mg·dL^−1^; LOA: −15.3 to +40.8 mg·dL^−1^). Slower rates of increase (1–2 mg·dL^−1^·min^−1^) yielded similar MARD values (13.5 ± 12.8%) but smaller bias (+4.9 ± 18.3 mg·dL^−1^). Accuracy was highest when glucose remained relatively stable (MARD: 10.5 ± 8.6%; bias: 0.0 ± 13.0 mg·dL^−1^; LOA: −25.4 to +25.4 mg·dL^−1^).

Both carbohydrate amount and glycaemic index were significant contributors to the peak post-ingestion, as expected from previous observations [20,21,22,23]. Fixed amounts of carbohydrate in varying fluid volumes (5%, 10%, and 20% solutions) appeared to produce similar peak glucose concentrations regardless of the carbohydrate concentration of the drink. MARD values were 12.5 ± 9.5% and 12.7 ± 9.5%, with a bias of 15.8 ± 18.4 mg/dL during hyperglycaemia (LOA: −20.3 to +52.0 mg·dL^−1^). This widening of the limits of agreement at higher glucose concentrations indicates greater dispersion and reduced reliability at the upper end of the range, a pattern also observed in recent CGM validation studies [8].

Greater glycaemic differences between [iG] and [BG] were observed during glucose declines. When glucose was falling rapidly (>2 mg/dL/min), MARD reached 20.4 ± 27.4%, and [iG] underestimated [BG] by −7.26 ± 27.1 mg/dL (LOA: −40.4 to +45.9 mg·dL^−1^). Even moderate declines (1–2 mg/dL/min) resulted in elevated MARD (17.0 ± 24.4%) and negative bias (−6.3 ± 18.8 mg/dL). Similarly, MARD in hypoglycaemia was elevated (18.5 ± 25%), with a consistent tendency for [iG] to underestimate [BG] (bias: −6.9 ± 14.6 mg·dL^−1^; LOA: −35.5 to +21.8 mg·dL^−1^).

The wider LOA observed during rapid changes and at glycaemic extremes suggests that transient physiological lag and sensor kinetics substantially influence the instantaneous alignment between compartments. Collectively, these data indicate that while overall bias remained relatively low, the limits of agreement were impacted substantially during rapid changes and at glycaemic extremes, suggesting that [iG] tracks directionality but cannot be considered interchangeable with [BG] on a point-by-point basis. The LOA observed in our study are consistent with those reported in similar cohorts, with comparable ranges of −20.7 to 42.8 mg/dL [24] and −25.9 to 67.0 mg/dL [18] reported elsewhere.

These findings in healthy individuals without diabetes confirm that differences between interstitial and blood glucose are most pronounced during fast-changing glycaemic states. CGM may misrepresent declines in glucose concentration. While this is unlikely to pose a substantial health risk in healthy populations, in a clinical context, this can be particularly hazardous for insulin-dependent individuals who rely on CGM for real-time decision-making [25]. This is especially important for those utilising an automated insulin delivery (AID) system, where insulin dosing decisions are made algorithmically based on interstitial glucose trends [26]. If a device underestimates [BG] during a declining phase, it may fail to alert users to an impending hypoglycaemic episode or prompt corrective carbohydrate ingestion based on a perceived low [iG] value, when blood glucose is not critically low. A confirmation via a self-monitoring blood glucose (SMBG) device might be advised for safety reasons under these circumstances, especially when symptoms do not match CGM trends [12].

For healthy or athletic populations, this inaccuracy has broader implications. In situations where athletes use CGM to guide fuelling during extended or high-intensity sessions, a false sense of urgency to consume carbohydrates, or a failure to respond to actual hypoglycaemia, could compromise performance or safety.

Together with prior findings, our data suggest CGM provides valuable trend information but should not be solely relied upon during times of rapid glucose change or in hypoglycaemia. Practitioners and users must interpret low [iG] values cautiously, considering glucose trajectory and context rather than absolute values alone.

### 4.2. Factors Influencing CGM Accuracy

Mean blood glucose is important in the clinical use of CGM as it is linked to HbA_1c_ over prolonged periods [27]. While mean [iG] and [BG] were generally similar over two hours, this masked discrepancies during post-peak declines. For example, the 20% carbohydrate trial showed a greater drop in post-peak [iG], lowering overall mean [iG]. While sensor kinetics and peripheral tissue uptake are the primary drivers of post-peak iG/BG divergence, the hyperosmolar nature of the 20% solution may have prolonged intestinal glucose absorption, contributing to the slower [BG] decline observed (1.0 vs. 1.6 mg/dL/min, *p* = 0.007). As gastric emptying was not measured, the relative contributions of these mechanisms cannot be fully disentangled. This highlights the limitations of summary metrics in capturing acute glucose dynamics. This observation warrants further exploration in clinical cohorts such as those with diabetes.

Glycaemia is generally considered to be relatively tightly controlled in healthy participants. The range of expected concentration deviations is much less than that experienced in diabetes. However, accuracy in our study was better than what was observed in other studies with healthy participants after acute feeding. Jin et al. [18] reported much greater MARDs during all glycaemic states and rates of change; however, in their study, CGM was compared against venous sampling, which typically yields lower glucose concentrations than capillary blood in the postprandial state due to peripheral glucose extraction [28], likely contributing to the greater MARD values observed. Moser et al. [19] also examined the accuracy of CGM in relation to glycaemic states in individuals with type 1 diabetes. In comparison to our data, accuracy (assessed by MARD) was slightly worse overall (13.5 ± 13.8 vs. 14.3%), during euglycaemia (12 ± 10.3 vs. 16%), and during hypoglycaemia (18.5 ± 25 vs. 31.6%), but it was slightly better during hyperglycaemia (12.7 ± 9.5 vs. 9.4%). It is worth noting that the range of deviation for each measure in our study was relatively high.

Some of the differences observed may relate to the time required for glucose to move from blood into the interstitial space [9]. Cellular uptake may also create another source of divergence from blood concentration [29]. While CGM algorithms attempt to compensate for this delay, the observed errors show that such filtering is not always sufficient to preserve accuracy [30], especially during rapid declines [31].

Falling glucose thus remains the most challenging condition for CGM accuracy, where tissue glucose uptake and sensor lag combine to produce the greatest divergence from blood glucose values.

### 4.3. Sensor Design and Algorithmic Filtering

CGM sensors produce an electrochemical signal susceptible to biological interference from factors including hydration, temperature, movement, and pressure. Time lag between blood and interstitial glucose is inherent, though modern CGMs use filtering algorithms and predictive modelling to smooth data, improve accuracy, and reduce artefacts [32]. While these advances have lowered MARD over time, they highlight the limits of how precisely sensors can reflect blood glucose instantaneously.

### 4.4. Practical Implications

This study demonstrates that CGM can reflect the general direction of blood glucose change in response to carbohydrate ingestion, but caution is required when interpreting values during rapidly changing or low glucose states [25]. Researchers and practitioners using CGM in acute non-clinical settings should be aware that common metrics such as mean glucose may not fully capture the dynamics of interest. Metrics based on the rate of change or relative trends may be more appropriate in these contexts. In applied settings, this is particularly relevant for athletes using CGM to guide fuelling decisions during competition or training, where the underestimation of a rapidly falling blood glucose could have consequences for both performance and safety. In addition, some caveats must be applied to the level of accuracy of displayed values. More chronic glycaemic investigations may also warrant the use of metrics which better reflect long-term glycaemia, such as time in range, given the limitations of short-term acute observations.

### 4.5. Strengths and Limitations

A key strength of this study was its randomised, within person, controlled design, which enabled direct comparisons across different carbohydrate formulations. The inclusion of a placebo trial helped to isolate beverage effects from natural glycaemic variability. A limitation was the CGM measurement floor of 54 mg/dL, which may have prevented detection of true minimum values and influenced some post-peak metrics. However, [iG] still measured lower than [BG] in several instances, suggesting that this limitation did not affect the main findings. One notable limitation is the sample size. Given the exploratory aim of characterising acute glycaemic responses and sensor behaviour, rather than detecting population-level effects, a sample size of ten participants was employed. Post hoc inspection indicated sufficient power to detect large within-subject differences (Cohen’s d ≈ 0.8–1.0), as observed for variables such as minimum glucose concentration and time to peak, where differences of 5–9 mg·dL^−1^ or 6–8 min were statistically significant (*p* < 0.05). However, for parameters such as peak and mean glucose concentrations or rates of change, between-compartment differences were typically small (<4 mg·dL^−1^) relative to their variability (SD ≈ 15–25 mg·dL^−1^), resulting in small effect sizes (d < 0.3) and correspondingly low power (<40%). Detecting such small differences with 80% power would require approximately 40–80 participants, which is impractical given the repeated-measures, crossover design with multiple laboratory visits. Thus, the chosen sample size represented a pragmatic balance between statistical rigour and logistical feasibility, consistent with the exploratory rather than definitive aims of this investigation.

## 5. Conclusions

This study aimed to assess the agreement between blood glucose ([BG]) and interstitial glucose ([iG]) responses following ingestion of carbohydrate-containing beverages differing in amount, concentration, and glycaemic index. While CGM-derived [iG] generally tracked the direction of [BG] changes, discrepancies emerged during periods of rapid glucose decline or hypoglycaemia. Overall, MARD (13.5 ± 13.8%) aligns with prior healthy individual data [11]. Accuracy was acceptable during stable or rising glucose but requires caution during declines to avoid underestimation and misinterpretation. In applied or research contexts, glucose trend direction and rate of change are essential complements to absolute [iG] values for meaningful interpretation.

## Figures and Tables

**Table 1:** Summary of interstitial [iG] and blood glucose [BG] measurements, and differences for all trials. * indicates a statistical difference between BG and iG *p* ≤ 0.05. † indicates where measurements were estimated due to values exceeding the measurement range. This estimate was made based on expected osmolarity (2 times the 10% beverage osmolarity) All data are displayed as the mean ± SD; *n* varies by condition (range 9–11); trials with interrupted CGM data continuity were excluded.

Amount of CHO	50 g		[BG] vs. [iG]	25 g		[BG] vs. [iG]	10 g		[BG] vs. [iG]
	iG	BG		iG	BG		iG	BG	
Peak (mg/dL)	158 ± 17	154 ± 23	*p* = 0.434	137 ± 17	133 ± 17	*p* = 0.496	107 ± 22	107 ± 14	*p* = 0.950
Mean (mg/dL)	104 ± 9	104 ± 13	*p* = 0.997	93 ± 6	94 ± 10	*p* = 0.690	82 ± 10	85 ± 8	*p* = 0.384
Min (mg/dL)	62 ± 7	59 ± 15	*p* = 0.633	62 ± 6	67 ± 4	*p* = 0.034 *	68 ± 8	72 ± 8	*p* = 0.129
Time to peak (mins)	37 ± 8	35 ± 10	*p* = 0.343	31 ± 5	23 ± 5	*p* = 0.033 *	29 ± 7	24 ± 8	*p* = 0.05 *
Time to minimum (mins)	98 ± 20	98 ± 34	*p* = 0.934	71 ± 25	85 ± 28	*p* = 0.442	72 ± 18	102 ± 20	*p* = 0.01 *
ROC [rest-peak] (mg/dL/min)	2.2 ± 0.6	2.3 ± 1.0	*p* = 0.772	1.9 ± 0.5	2.3 ± 0.8	*p* = 0.233	1.2 ± 0.7	1.3 ± 0.7	*p* = 0.689
ROC [peak to minimum] (mg/dL/min)	1.8 ± 0.4	1.3 ± 0.4	*p* = 0.063	1.6 ± 0.4	1.3 ± 0.8	*p* = 0.325	1.2 ± 0.8	0.6 ± 0.3	*p* = 0.019 *
Concentration of Solution	5% 720 ± 0 mOsm.kgH_2_O		[BG] vs. [iG]	10% 1270 ± 0 mOsm.kgH_2_O		[BG] vs. [iG]	20% 2540 † ± 0 mOsm.kgH_2_O		[BG] vs. [iG]
	iG	BG		iG	BG		iG	BG	
Peak (mg/dL)	137 ± 17	133 ± 17	*p* = 0.496	134 ± 18	133 ± 16	*p* = 0.928	138 ± 26	137 ± 31	*p* = 0.596
Mean (mg/dL)	93 ± 6	94 ± 10	*p* = 0.690	94 ± 11	94 ± 10	*p* = 0.913	94 ± 13	101 ± 16	*p* = 0.03 *
Min (mg/dL)	63 ± 6	68 ± 4	*p* = 0.019 *	64 ± 5	64 ± 13	*p* = 0.983	60 ± 3	69 ± 8	*p* = 0.001*
Time to peak (mins)	31 ± 5	23 ± 5	*p* = 0.003 *	38 ± 7	33 ± 11	*p* = 0.05 *	36 ± 7	35 ± 9	*p* = 0.780
Time to minimum (mins)	71 ± 25	85 ± 28	*p* = 0.442	87 ± 15	94 ± 26	*p* = 0.237	90 ± 22	84 ± 44	*p* = 0.671
ROC [rest-peak] (mg/dL/min)	1.9 ± 0.5	2.3 ± 0.8	*p* = 0.145	1.4 ± 0.6	1.8 ± 0.8	*p* = 0.092	1.7 ± 0.7	1.7 ± 0.8	*p* = 0.742
ROC [peak to minimum] (mg/dL/min)	1.6 ± 0.4	1.3 ± 0.8	*p* = 0.325	1.4 ± 0.4	1.3 ± 0.8	*p* = 0.607	1.6 ± 0.7	1.0 ± 0.6	*p* = 0.007 *
High vs. Low GI	DEX (High)		[BG] vs. [iG]	ISO (Low)		[BG] vs. [iG]	Placebo 500 mL		[BG] vs. [iG]
	iG	BG		iG	BG		iG	BG	
Peak	158 ± 17	154 ± 23	*p* = 0.434	113 ± 11	113 ± 17	*p* = 0.972	86 ± 8	84 ± 12	*p* = 0.542
Mean	104 ± 9	104 ± 13	*p* = 0.997	93 ± 6	95 ± 8	*p* = 0.320	82 ± 8	77 ± 10	*p* = 0.176
Min	62 ± 7	59 ± 15	*p* = 0.633	76 ± 7	78 ± 10	*p* = 0.539	77 ± 8	69 ± 11	*p* = 0.035 *
Time to peak	37 ± 8	35 ± 10	*p* = 0.343	43 ± 10	42 ± 15	*p* = 0.780	44 ± 35	25 ± 18	*p* = 0.070
Time to minimum	98 ± 20	98 ± 34	*p* = 0.934	72 ± 47	92 ± 36	*p* = 0.159	70 ± 40	89 ± 36	*p* = 0.236
ROC [rest-peak] (mg/dL/min)	2.2 ± 0.6	2.3 ± 1.0	*p* = 0.772	0.7 ± 0.3	0.8 ± 0.1	*p* = 0.600	0.1 ± 0.3	0.2 ± 0.4	*p* = 0.670
ROC [peak to minimum] (mg/dL/min)	1.8 ± 0.4	1.3 ± 0.4	*p* = 0.063	0.7 ± 0.4	0.6 ± 0.4	*p* = 0.432	−0.0 ± 0.4	0.4 ± 0.3	*p* = 0.057

**Table 2:** Displays the percentage of Mean absolute relative difference (MARD) between blood and interstitial glucose concentration in each scenario.

	MARD % (95% CI)	Bland Altman Bias mg/dL (95% LOA)
Overall (n = 923)	13.5 ± 13.8% (12.6, 14.4%)	1.1 ± 15.8 (−29.9, 32)
Hyperglycaemia	12.7 ± 9.5% (9.7, 15.6%)	15.8 ± 18.4 (−20.3, 52.0)
Euglycaemia	12.5 ± 10.3% (11.8, 13.3%)	1.7 ± 15.1 (−27.8, 31.2)
Hypoglycaemia	18.5 ± 25% (14.3, 22.7%)	−6.9 ± 14.6 (−35.5, 21.8)
Rising quickly (>2 mg/dL/min)	13.8 ± 13.1% (11.8, 15.8%)	12.9 ± 14.3 (−15.3, 40.8)
Rising (1–2 mg/dL/min)	13.5 ± 12.8% (10.7, 16.2%)	4.9 ± 18.3 (−31.0, 40.7)
Stable (<1 mg/dL/min)	10.5 ± 8.6% (9.5, 11.5%)	0.00 ± 13 (−25.4, 25.4)
Falling (1–2 mg/dL/min)	17.0 ± 24.4% (13.5, 20.5%)	−6.3 ± 18.8 (−43.1, 30.4)
Falling quickly (>2 mg/dL/min)	20.4 ± 27.4% (11.9, 28.9%)	−7.26 ± 27.1 (−40.4, 45.9)

## Data Availability

The original contributions presented in this study are included in the article/Supplementary Material. Further inquiries can be directed to the corresponding author.

## Supplementary Materials

The following supporting information can be downloaded at: https://www.mdpi.com/article/10.3390/nu18122033/s1.
