# Supplementary material for: A Comparison of the Interstitial and Blood Glucose Responses Following Consumption of Different Carbohydrate-Containing Beverages in Humans: A Randomised Controlled Trial
Source: Nutrients. 2026 Jun 22;18(12):2033. doi: 10.3390/nu18122033 (PMC13306023; doi:10.3390/nu18122033)
Supplement: Supplementary file 1 [file nutrients-18-02033-s001.zip › Supplemental table 1.pdf]

| CHO amount | 50g [iG] | 50g [BG] | [BG] vs. [iG] | 50g [iG] Offset | 50g [BG] offset | [BG] vs. [iG] | 25g [iG] | 25g [BG] | [BG] vs. [iG] | 25g [iG] offset | 25g [iG] Offset | [BG] vs. [iG] | 10g [iG] | 10g [BG] | [BG] vs. [iG] | 10g [iG] offset | 10g [BG] offset | [BG] vs. [iG] |
|---|---|---|---|---|---|---|---|---|---|---|---|---|---|---|---|---|---|---|
| **Area of Positive AUC** | 2732 | 2564 | p=0.743 | 2590 | 2757 | p=0.789 | 1561 | 1406 | p=0.661 | 1466 | 1498 | p=0.934 | 913 | 730 | p=0.513 | 818 | 1084 | p=458 |
| **Std. Error** | 339 | 383 | | 415 | 442 | | 227 | 271 | | 216 | 319 | | 234 | 152 | | 215 | 286 | |
| **95% Confidence Interval** | 2067 to 3397 | 1812 to 3315 | | 1777 to 3403 | 1891 to 3622 | | 1117 to 2005 | 875 to 1937 | | 1042 to 1890 | 873 to 2122 | | 454 to 1372 | 432 to 1028 | | 398 to 1239 | 524 to 1645 | |
| **Area of Negative AUC** | 408 | 468 | p=0.875 | 387 | 204 | p=0.598 | 549 | 529 | p=0.938 | 606 | 187 | p=0.267 | 0 | 277 | p=0.081 | 0 | 0 | NA |
| **Std. Error** | 243 | 295 | | 230 | 259 | | 205 | 151 | | 294 | 235 | | 0 | 158 | | 0 | 0 | |
| **95% Confidence Interval** | 0.0 to 884 | 0.0 to 1045 | | 0.0 to 838 | 0.0 to 710 | | 147 to 950 | 234 to 824 | | 30 to 1182 | 0.0 to 647 | | 0.0 to 0.0 | 0.0 to 587 | | 0.0 to 0.0 | 0.0 to 0.0 | |
| **Total Area** | 3140 | 3032 | p=0.743 | 2977 | 2960 | p=0.981 | 2110 | 1935 | p=0.688 | 2078 | 1685 | p=0.468 | 947 | 1007 | p=0.868 | 874 | 1084 | p=0.587 |
| **Std. Error** | 417 | 483 | | 474 | 512 | | 305 | 310 | | 368 | 396 | | 285 | 219 | | 247 | 286 | |
| **95% Confidence Interval** | 2322 to 3958 | 2084 to 3979 | | 2048 to 3907 | 1957 to 3963 | | 1511 to 2709 | 1327 to 2542 | | 1356 to 2800 | 909 to 2461 | | 390 to 1505 | 577 to 1436 | | 389 to 1358 | 524 to 1645 | |
| **Concentration** | **5% [iG]** | **5% [BG]** | **[BG] vs. [iG]** | **5% [iG] offset** | **5% [BG] Offset** | **[BG] vs. [iG]** | **10% [iG]** | **10% [BG]** | **[BG] vs. [iG]** | **10% [iG] offset** | **10% [BG] Offset** | **[BG] vs. [iG]** | **20% [iG]** | **20% [BG]** | **[BG] vs. [iG]** | **20% [iG] offset** | **20% [BG] Offset** | **[BG] vs. [iG]** |
| **Area of Positive AUC** | 1561 | 1406 | p=0.662 | 1466 | 1498 | p=0.934 | 1491 | 1550 | p=0.889 | 1455 | 1803 | p=0.460 | 1955 | 2095 | p=0.460 | 1915 | 2348 | p=0.531 |
| **Std. Error** | 227 | 271 | | 216 | 319 | | 272 | 321 | | 268 | 386 | | 390 | 456 | | 423 | 545 | |
| **95% Confidence Interval** | 1117 to 2005 | 875 to 1937 | | 1042 to 1890 | 873 to 2122 | | 959 to 2024 | 920 to 2181 | | 929 to 1980 | 1047 to 2560 | | 1189 to 2720 | 1202 to 2989 | | 1085 to 2745 | 1281 to 3416 | |
| **Area of Negative AUC** | 549 | 529 | p=0.938 | 606 | 187 | p=0.267 | 608 | 505 | p=0.696 | 662 | 221 | p=0.228 | 437 | 272 | p=0.816 | 415 | 0 | **p=0.016*** |
| **Std. Error** | 205 | 151 | | 294 | 235 | | 162 | 207 | | 231 | 282 | | 184 | 123 | | 171 | 0 | |
| **95% Confidence Interval** | 147 to 950 | 234 to 824 | | 30 to 1182 | 0.0 to 647 | | 291 to 924 | 100 to 911 | | 210 to 1114 | 0.0 to 773 | | 77 to 797 | 30 to 514 | | 80 to 750 | 0.0 to 0.0 | |
| **Total Area** | 2110 | 1935 | p=0.688 | 2078 | 1685 | p=0.468 | 2099 | 2056 | p=0.931 | 2117 | 2024 | p=0.876 | 2392 | 2367 | p=0.970 | 2336 | 2404 | p=0.926 |
| **Std. Error** | 305 | 310 | | 368 | 396 | | 316 | 382 | | 354 | 478 | | 432 | 472 | | 459 | 565 | |
| **95% Confidence Interval** | 1511 to 2709 | 1327 to 2542 | | 1356 to 2800 | 909 to 2461 | | 1479 to 2719 | 1306 to 2805 | | 1423 to 2810 | 1087 to 2961 | | 1546 to 3238 | 1441 to 3293 | | 1436 to 3237 | 1296 to 3512 | |
| **Glycaemic Index** | **DEX [iG]** | **DEX [BG]** | **[BG] vs. [iG]** | **DEX [iG] Offset** | **DEX [BG] offset** | **[BG] vs. [iG]** | **ISO [iG]** | **ISO [BG]** | **[BG] vs. [iG]** | **ISO [iG] offset** | **ISO [BG] offset** | **[BG] vs. [iG]** | | | | | | |
| **Area of Positive AUC** | 2732 | 2564 | p=0.743 | 2590 | 2757 | p=0.789 | 857 | 1019 | p=0.649 | 837 | 1421 | p=0.270 | | | | | | |
| **Std. Error** | 339 | 383 | | 415 | 442 | | 256 | 246 | | 307 | 431 | | | | | | | |

| | | | | | | | | | | | | |
|---|---|---|---|---|---|---|---|---|---|---|---|---|
| **95% Confidence Interval** | 2067 to 3397 | 1812 to 3315 | | 1777 to 3403 | 1891 to 3622 | | 355 to 1360 | 536 to 1501 | | 235 to 1440 | 575 to 2266 | |
| **Area of Negative AUC** | 408 | 468 | p=0.875 | 387 | 204 | p=0.598 | 18 | 68 | p=0.718 | 31 | 0 | p=0.363 |
| **Std. Error** | 243 | 295 | | 230 | 259 | | 22 | 137 | | 34 | 0 | |
| **95% Confidence Interval** | 0.0 to 884 | 0.0 to 1045 | | 0.0 to 838 | 0.0 to 710 | | 0.0 to 60 | 0.0 to 336 | | 0.0 to 98 | 0.0 to 0.0 | |
| **Total Area** | 3140 | 3032 | p=0.743 | 2977 | 2960 | p=0.981 | 889 | 1087 | p=0.610 | 874 | 1421 | p=0.306 |
| **Std. Error** | 417 | 483 | | 474 | 512 | | 267 | 281 | | 314 | 431 | |
| **95% Confidence Interval** | 2322 to 3958 | 2084 to 3979 | | 2048 to 3907 | 1957 to 3963 | | 366 to 1413 | 535 to 1638 | | 259 to 1489 | 575 to 2266 | |

**Supplemental Table S1.** Glucose responses (area under the curve, AUC) to beverages varying in carbohydrate amount, concentration, and glycaemic index, comparing interstitial glucose [iG] and blood glucose concentrations [BG]. Absolute AUC values and placebo-adjusted (offset). Positive AUC represents glucose excursions above baseline, while negative AUC reflects dips below baseline. Total AUC represents net glucose excursion. * indicates a statistical difference between iG & BG $p \leq 0.05$.
