# Supplementary material for: A Comparison of the Interstitial and Blood Glucose Responses Following Consumption of Different Carbohydrate-Containing Beverages in Humans: A Randomised Controlled Trial
Source: Nutrients. 2026 Jun 22;18(12):2033. doi: 10.3390/nu18122033 (PMC13306023; doi:10.3390/nu18122033)
Supplement: Supplementary file 1 [file nutrients-18-02033-s001.zip › Supplemental Table 2.pdf]

Carbohydrate amount								
	50g		25g		10g		PLAC	
**Δ**	**Hct**	**Hb**	**Hct**	**Hb**	**Hct**	**Hb**	**Hct**	**Hb**
**Baseline**	43±4	144±11	43±3	137±11	42±3	146±11	44±2	146±7
**30min**	43±3	140±7	43±3	144±9	43±2	146±8	44±3	148±9
**60min**	43±4	141±8	43±3	145±5	44±2	145±8	44±3	148±8
**90min**	44±4	148±6	43±3	**149±10**	43±3	146±7	44±2	148±10
**120min**	43±4	147±11	43±3	**151±7**	44±3	145±8	44±2	145±14

Concentration of solution								
	5%		10%		20%		PLAC	
**Δ**	**Hct**	**Hb**	**Hct**	**Hb**	**Hct**	**Hb**	**Hct**	**Hb**
**Rest**	43±3	137±11	42±3	141±8	43±3	143±8	44±2	146±7
**30 mins**	43±3	144±9	43±3	144±10	43±3	145±11	44±3	148±9
**60 mins**	43±3	145±5	43±3	141±9	43±3	144±8	44±3	148±8
**90 mins**	43±3	**149±10**	44±3	144±10	44±3	141±11	44±2	148±10
**120 mins**	43±3	**151±7**	44±3	148±7	44±3	147±12	44±2	145±14

Glycaemic index						
	DEX		ISO		PLAC	
**Δ**	**Hct**	**Hb**	**Hct**	**Hb**	**Hct**	**Hb**
**Rest**	43±4	144±11	42±3	142±7	44±2	146±7
**30**	43±3	140±7	43±2	144±6	44±3	148±9
**60**	43±4	141±8	43±2	151±6	44±3	148±8
**90**	44±4	148±6	44±2	144±8	44±2	148±10
**120**	43±4	147±11	43±2	150±6	44±2	145±14

Supplemental Table S2. Haemoglobin (Hb) (g/L) and haematocrit (Hct) (%) changes from rest (baseline) and thereafter at 30, 60, 90 and 120 min timepoints after consuming a beverage with carbohydrates with different amounts of carbohydrate (**50g, 25g and 10g**), different concentrations (**5%, 10% and 20%**) and different glycaemic index (**DEX** and **ISO**) compared to a Placebo (**PLAC**) in a fixed volume of water (500mls). Values in bold indicate a difference from resting values. * indicates a difference between **DEX** and **ISO.** Data is displayed as mean±SD, p≤0.05.
