# Supplementary material for: A Comparison of the Interstitial and Blood Glucose Responses Following Consumption of Different Carbohydrate-Containing Beverages in Humans: A Randomised Controlled Trial
Source: Nutrients. 2026 Jun 22;18(12):2033. doi: 10.3390/nu18122033 (PMC13306023; doi:10.3390/nu18122033)
Supplement: Supplementary file 1 [file nutrients-18-02033-s001.zip › Supplemental Table 3.pdf]

Carbohydrate amount				
**PV%Δ**	**50g**	**25g**	**10g**	**PLAC**
**Rest**	-	-	-	-
**30min**	3.4±9.2	-4.8±6.7	-1.2±4.8	-1.2±7.3
**60min**	-0.2±9.3	-1.4±5.3	0.2±6.8	-0.4±8.1
**90min**	-5.8±3.2	-2.3±6.5	7.3±20.1	0.2±7.8
**120min**	2±7.7	-1.1±7.1	-4.5±14.6	2.2±9.1
**Concentration of solution**				
**PV%Δ**	**5%**	**10%**	**20%**	**PLAC**
**Rest**	-	-	-	-
**30 mins**	3.4±9.2	-8.0±15.3	-0.8±10.7	-1.2±7.3
**60 mins**	-0.2±9.3	2.4±6.5	1.4±7.2	-0.4±8.1
**90 mins**	-5.8±3.2	-2.8±7.5	1.3±9.0	0.2±7.8
**120 mins**	2±7.7	-2.8±8.8	-3.6±6.5	2.2±9.1
**Glycaemic index**				
**PV%Δ**	**DEX**	**ISO**	**PLAC**	
**Baseline**	-	-	-	
**30**	3.4±9.2	-2.1±8.4	-1.2±7.3	
**60**	-0.2±9.3	-4.1±6.9	-0.4±8.1	
**90**	-5.8±3.2	3.3±10	0.2±7.8	
**120**	2±7.7	-3.1±5.0	2.2±9.1	

Supplemental Table S3. Plasma volume changes from rest (baseline) and thereafter at 30, 60, 90 and 120min timepoints after consuming a beverage with carbohydrates with different amounts of carbohydrate (**50g, 25g and 10g**), different concentrations (**5%, 10% and 20%**) and different glycaemic index (**DEX** and **ISO**) compared to a Placebo (**PLAC**) in a fixed volume of water (500mls). Data is displayed as mean±SD, $p \leq 0.05$.
